# Characterization and Antimicrobial Susceptibility Patterns of Clinically Significant Non-fermenting Gram-Negative Bacilli Isolated From Clinical Specimens

**DOI:** 10.7759/cureus.116023

**Published:** 2026-09-09

**Authors:** Dhanashree R Chavan, Ravindra V Shinde, Satish R Patil

**Affiliations:** 1 Department of Microbiology, Krishna Institute of Medical Sciences, Krishna Vishwa Vidyapeeth (Deemed to be University), Karad, IND

**Keywords:** acinetobacter baumannii, antimicrobial susceptibility pattern, non-fermenting gram-negative bacilli, pseudomonas aeruginosa, stenotrophomonas maltophilia

## Abstract

Background

Non-fermenting Gram-negative bacilli (NFGNB) have emerged as significant opportunistic pathogens associated with infections in healthcare settings and multidrug resistance. Their ability to persist in hospital settings and develop resistance to multiple antimicrobial agents poses a serious challenge to patient management. The current study aimed to determine the distribution of NFGNB isolates across different clinical samples and to evaluate their antimicrobial susceptibility and multidrug resistance patterns.

Materials and methods

A cross-sectional investigation was carried out on 127 NFGNB that were isolated from clinical samples received in the Department of Microbiology Laboratory of a tertiary care hospital. Antimicrobial susceptibility testing was performed in accordance with the Clinical and Laboratory Standards Institute (CLSI) guidelines, and the isolates were identified using conventional microbiological techniques. Demographic characteristics, specimen distribution, bacterial profile and multidrug resistance patterns were studied.

Results

A total of 127 NFGNB isolates were recovered, of which 86 (67.72%) were from male patients and 41 (32.28%) were from female patients. The highest prevalence of NFGNB was observed in the 61-70-year age group, with 26 (20.47%) isolates, followed by the 51-60-year age group, with 23 (18.11%) isolates. Endotracheal (ET) aspirates were the most common specimen source, accounting for 34 (26.77%) isolates, followed by pus/discharge/wound swabs with 27 (21.26%) and urine with 26 (20.47%) isolates. 88 (69.29%) of the NFGNB isolates were obtained from patients admitted to the intensive care unit, whereas 39 (30.71%) were isolated from patients admitted to the wards. *Pseudomonas aeruginosa* was the most common isolate, accounting for 61 (48.03%) isolates, followed by *Acinetobacter baumannii* with 53 (41.73%) and *Stenotrophomonas maltophilia* with 13 (10.24%) isolates. *A. baumannii* and *P. aeruginosa* were highly carbapenem-resistant and fluoroquinolones and β-lactam antibiotics.

Conclusion

*A. baumannii* and *P. aeruginosa* were the predominant NFGNB isolates and exhibited a high rate of multidrug resistance, whereas *Stenotrophomonas maltophilia* remained susceptible to the antibiotics tested. Continuous surveillance of antibiotic resistance patterns, implementation of prevention and management of infections, and policies for antimicrobial stewardship are crucial to curb the dissemination of multidrug-resistant NFGNB and optimize patient outcomes.

## Introduction

The taxonomically distinct class of aerobic bacteria, non-spore-forming bacilli known as non-fermenting Gram-negative bacteria (NFGNB), either do not use glucose as a fuel source or metabolize it oxidatively [1]. In the environment, these organisms are found all over the world as saprophytes, although certain species may also exist as human commensals in the intestinal tract. NFGNB are roughly 15% of all bacterial isolates, according to estimates recovered in medical microbiology laboratories. However, the extensive and empirical application of antibiotics has led to the emergence of NFGNB as recognized healthcare-associated infectious agents in recent years [2]. In nature, these organisms are extensively dispersed and can survive in various hospital reservoirs such as medical devices, parenteral fluids, distilled water, and sinks [3]. They exhibit intrinsic resistance to many disinfectants. They are often found in hospitalized patients, on the hands of healthcare workers, and in the hospital environment, thereby posing a major challenge to infection control and prevention strategies [4].

The occurrence of multidrug-resistant (MDR) organisms is increasing, and this signifies a serious worldwide public health issue. To address the increasing problem of antimicrobial resistance, the World Health Organization (WHO) developed the Priority Pathogens List to identify bacterial species that need attention and the creation of new therapeutic strategies. Among these pathogens, particular emphasis is placed on MDR Gram-negative bacilli (GNB). Notably, carbapenem-resistant (CR) *Acinetobacter baumannii* and CR *Pseudomonas aeruginosa *are two NFGNB on this priority list that are of particular concern. Among the NFGNB isolated from clinical specimens, *Pseudomonas *species are the most frequently encountered, followed by *Acinetobacter *species, *Burkholderia *species and *Stenotrophomonas maltophilia* [3,4].

NFGNB are important healthcare-associated pathogens responsible for a variety of serious infections, such as urinary tract infections, lower respiratory tract infections, septicemia, meningitis, surgical site infections, and skin infections of the soft tissues. An increased prevalence of NFGNB, particularly species of *Pseudomonas *and *Acinetobacter*, has been reported in ICUs, probably due to widespread use of invasive medical devices and prolonged hospitalization [3]. The pathogenic role of NFGNB has been thoroughly recorded, especially among patients who are currently hospitalized or have been recently hospitalized. Immunocompromised states such as oncology and organ transplant patients, cystic fibrosis, traumatic injuries, mechanical ventilation and urinary catheterization pose a risk for NFGNB infections [4].

The WHO has listed antimicrobial drug resistance as one of the top 10 global public health threats that humanity faces and calls for urgent action to achieve the Sustainable Development Goals. The increasing resistance of NFGNB, particularly *Acinetobacter *spp., to antibacterial agents has become a major global health concern. CR *Acinetobacter *spp. is listed as “the most urgent antimicrobial resistance threat,” and MDR *P. aeruginosa* is a “serious threat” according to the Centers for Disease Control and Prevention (CDC, USA) [3,5].

Although carbapenems were once regarded as the drugs of last resort for the management of NFGNB infections, the advent of CR-NFGNB has been increasingly observed throughout the world. These are highly adaptable organisms, coupled with their diverse intrinsic and acquired resistance mechanisms, enabling them to resist several major classes of antimicrobial agents, leading to few treatment options available. This antimicrobial resistance contributes to treatment failure, prolonged hospitalization, increased mortality, and higher healthcare expenditures [2,6]. In view of these concerns, this work was carried out to determine the frequency of NFGNB and their patterns of antibiotic resistance in a tertiary care facility.

## Materials and methods

Study design and sample size

This descriptive cross-sectional study was conducted at Krishna Charitable Hospital and Medical Research Centre, Karad. The ethical clearance for the study was approved by the Institutional Ethics Committee of Krishna Vishwa Vidyapeeth (Deemed to be University) (approval No: KVV/IEC/05/2024). Written informed consent was obtained from all study participants.

The number of participants included in the study was determined based on the previously reported prevalence of 49% by Maniyan G, Vedachalam D, and Chinnusamy N [7]. Using this prevalence, the study sample size was derived by applying the standard formula, which is n = 4pq/L^2^, where p (prevalence) = (0.49), q = 1-p = (0.51), and L = denotes the allowable error (precision), which was taken as 0.10. As a result, the sample size is 4x0.49x0.51/(0.10)^2^ = 99.96 (≈100) culture-positive isolates of NFGNB. Thus, based on the prevalence reported by Maniyan G, Vedachalam D, and Chinnusamy N, the minimum required sample size was calculated to be 100 [7].

Formula: n = 4pq/L^2^

where p = 0.49, q = 0.51, and L = 0.10 (allowable error).

n = [4 × 0.49 × 0.51]/(0.10)^2^ = 99.96 ≈100

Inclusion and exclusion criteria

All clinically significant NFGNB obtained from diverse clinical specimens, including urine, pus, blood, sputum, CSF, pleural, ascitic, and peritoneal fluid, and other relevant samples received from patients belonging to all age groups and both genders, were included in the study. Duplicate isolates obtained from the same patient during the same episode of infection were excluded.

Sample collection and processing

The study included all clinical specimens, such as urine, sputum, pus, blood, body fluids, endotracheal tube aspirate, and other relevant samples, which were obtained using aseptic techniques from all age groups. All specimens were collected in sterile containers and processed according to standard microbiological protocols.

Isolation and Identification of Isolates

Clinical specimens were subjected to direct microscopic examination using standard microbiological procedures, including wet mount preparation and Gram staining. Clinical specimens were inoculated onto appropriate culture media, including Blood agar, MacConkey agar, and Chocolate agar. All culture plates were incubated aerobically at 37°C for 18-24 hours. Preliminary identification of isolates was based on colony morphology, pigmentation, lactose fermentation characteristics, Gram staining, and routine biochemical characteristics following standard microbiological procedures.

A biochemical panel comprising catalase, oxidase, pigment production on nutrient agar, indole production, citrate utilization, a test for urease production, TSI agar, the Hugh-Leifson oxidative-fermentative (O/F) test, the nitrate reduction test, the decarboxylase test, and O/F utilization of sugars like lactose and mannitol completed the identification.

Antimicrobial Susceptibility Testing (AST)

Antibiotic susceptibility of the isolate was assessed using the Kirby-Bauer disc diffusion method following CLSI 2024 guidelines [8]. Antibiotic susceptibility testing was carried out using the following antibiotics: piperacillin-tazobactam (100/10 µg), ceftazidime (30 µg), cefepime (30 µg), aztreonam (30 µg), imipenem (10 µg), meropenem (10 µg), ciprofloxacin (5 µg), levofloxacin (5 µg), amikacin (30 µg), norfloxacin (10 µg), doxycycline (30 µg), minocycline (30 µg), gentamicin (10 µg), tetracycline (30 µg), and trimethoprim-sulfamethoxazole (1.25/23.75 µg). The strain of *P. aeruginosa *ATCC 27853 was used as the quality control. The diameter of the zones of inhibition was measured, and the results of susceptibility testing were interpreted according to CLSI 2024 guidelines.

Statistical analysis

This was a descriptive cross-sectional study; only descriptive statistics (frequency and percentage) were applied for analyzing data, and no inferential statistical tests were applied.

## Results

The study included a total of 127 samples that were processed. Considerable differences were noted in the age-wise distribution across the various age groups. The highest proportion was noted in the 61-70 years age group, with 26 (20.47%) cases, followed by the 51-60 years age group, with 23 (18.11%) cases. The 41-50 years and 31-40 years age groups accounted for 16 (12.60%) and 15 (11.81%) cases, respectively, while the 21-30 years age group accounted for 13 (10.24%) cases. The proportion was lower in the 71-80 years, 81-90 years, and 11-20 years age groups, with 12 (9.45%), 10 (7.87%), and 9 (7.09%) cases, respectively. The age groups of 91-100 years and 0-10 years showed the lowest proportions, with two (1.57%) and one (0.79%) instances, respectively. These observations suggest that the burden was highest in the 51-70 age group, with a steady decrease in prevalence in the younger and older age groups (Figure 1).

**Figure 1:**
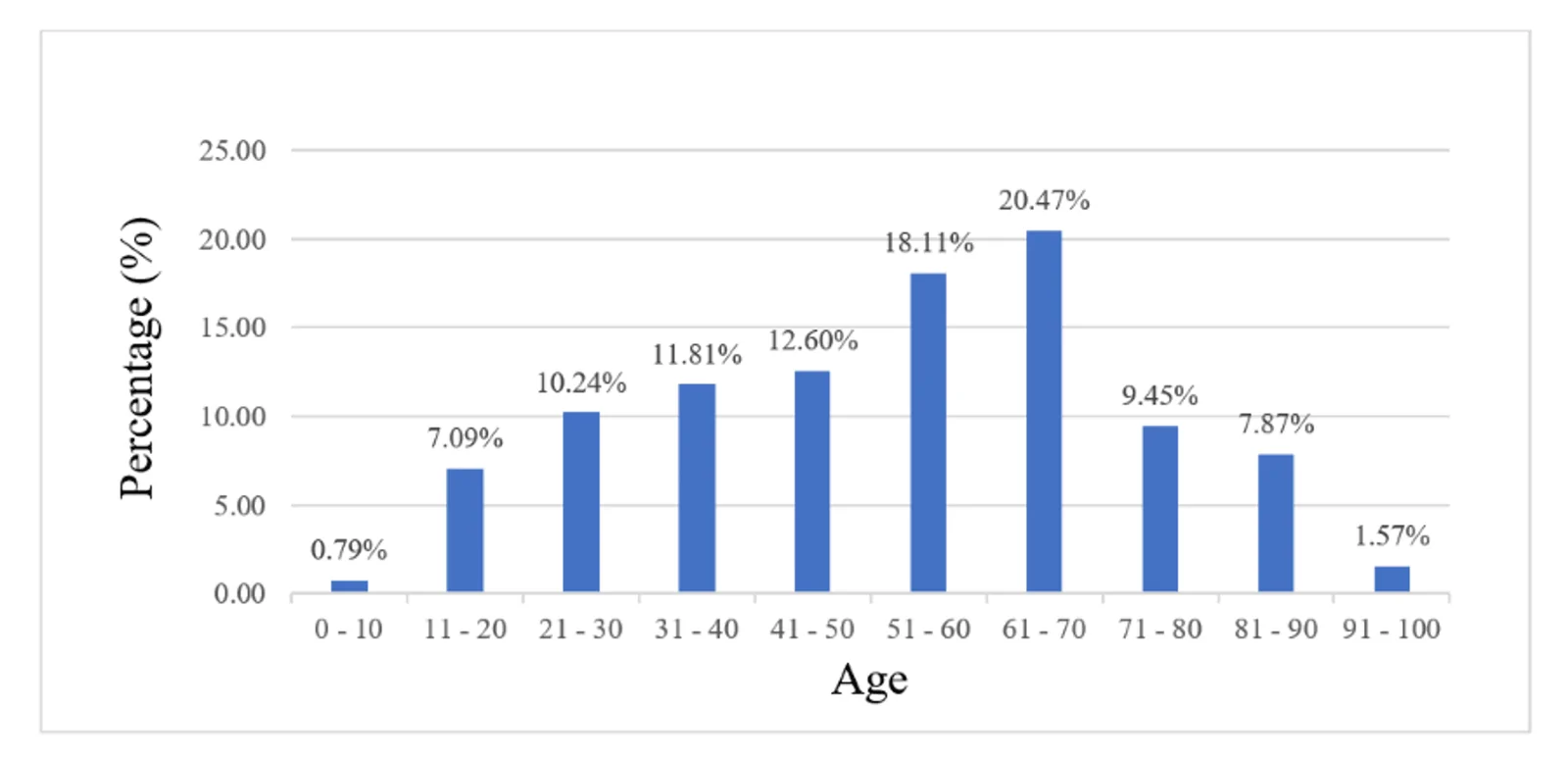
Age-wise distribution of non-fermenting Gram-negative bacilli (NFGNB).

Out of 127 isolates, males were shown to predominate with 86 isolates (67.72%) and 41 isolates (32.28%) from male and female patients, respectively. These data indicate that the study population has a higher rate of infection in males than in females (Figure 2).

**Figure 2:**
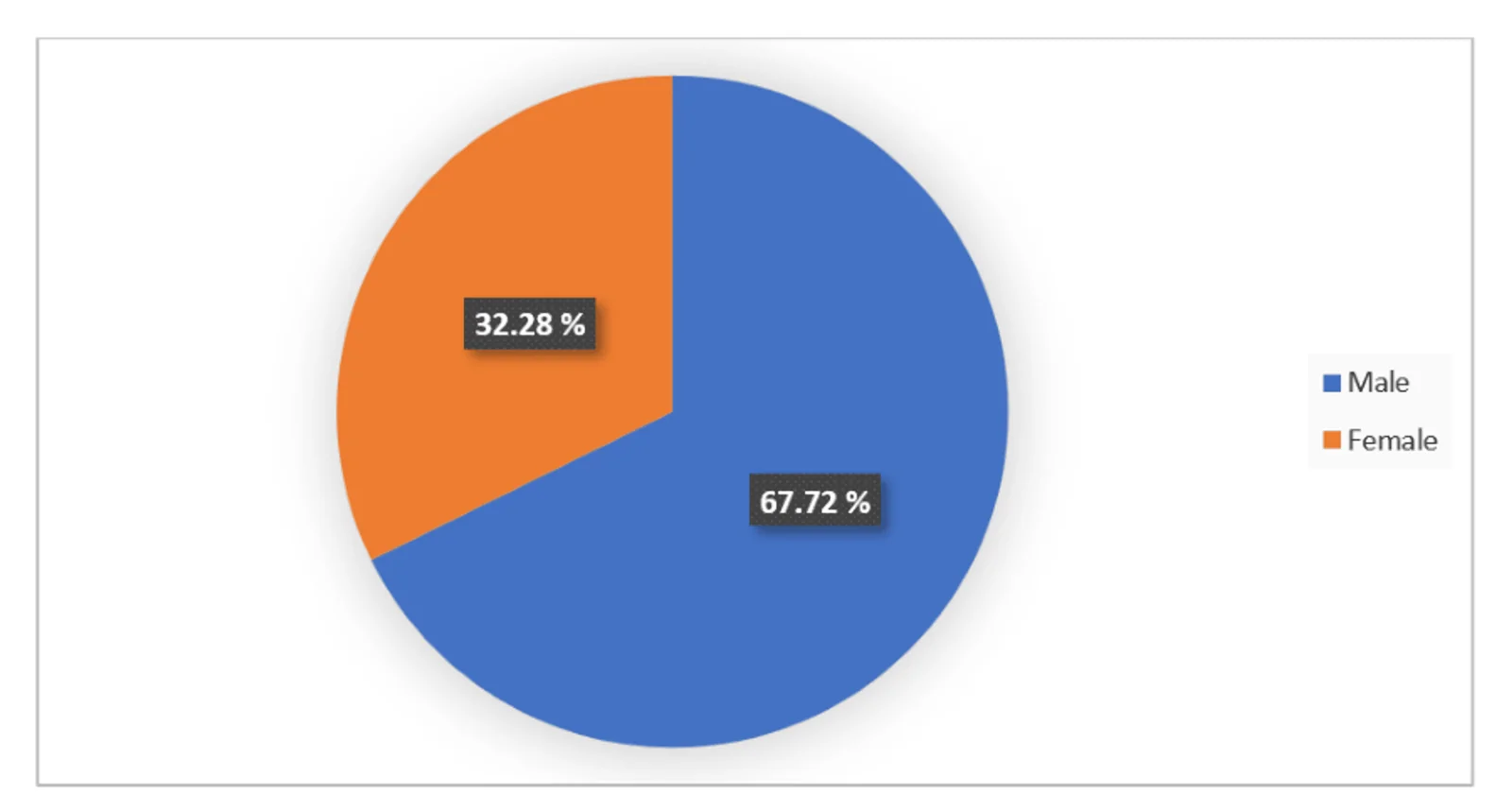
Gender-wise distribution of NFGNB.

*P. aeruginosa* was shown to be the most commonly isolated organism, with 61 isolates (48.03%) among the 127 isolates recovered in the current study. *A. baumannii* was the second most prevalently isolated organism (53 isolates, 41.73%). The least often isolated organisms were *S. maltophilia* (10.24%) with 13 isolates. The most prevalent was *P. aeruginosa* among the isolates, with *A. baumannii* not far behind, and *S. maltophilia* constituting a smaller proportion of all isolates (Figure 3).

**Figure 3:**
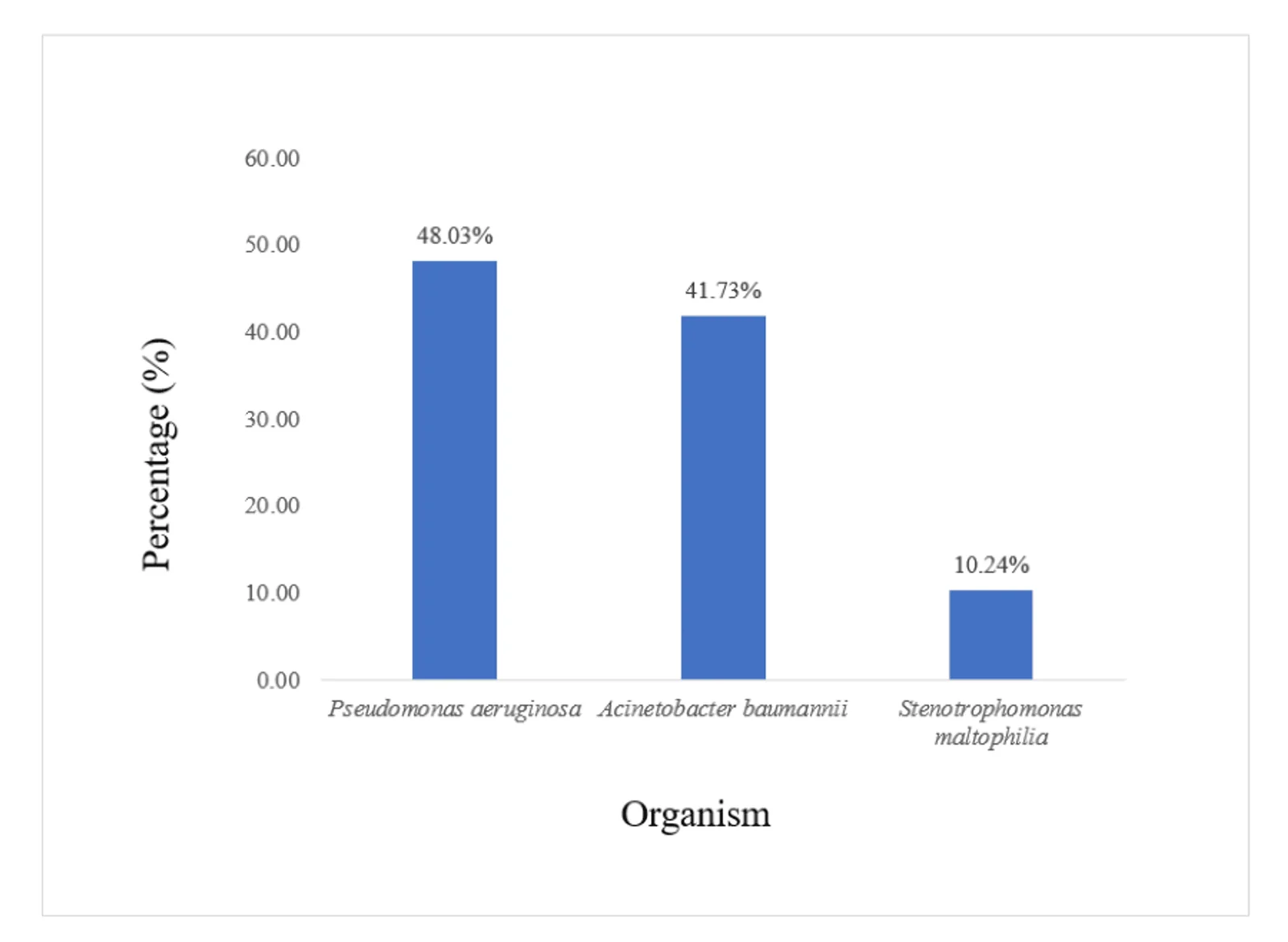
Organism-wise distribution of NFGNB among clinical isolates.

A total of 127 isolates were analyzed according to specimen type. Endotracheal aspirates (ETA) showed the maximum percentage of isolates, 34 (26.77%), followed by pus/discharge/wound swabs, 27 (21.26%), and urine samples, 26 (20.47%). Sputum specimens accounted for 20 (15.75%) isolates, while blood specimens accounted for 10 (7.87%) isolates. Lower proportions were observed in fluid samples, with 5 (3.94%) isolates, and tip cultures, with 3 (2.36%) isolates. The lowest number of isolates were obtained from high vaginal swab samples and bronchoalveolar lavage (BAL), each of which obtained 1 (0.79%) isolate. Overall, the major sources of isolates in the conducted study population were respiratory specimens (ETA and sputum) together with pus/discharge/wound swabs and urine samples (Figure 4).

**Figure 4:**
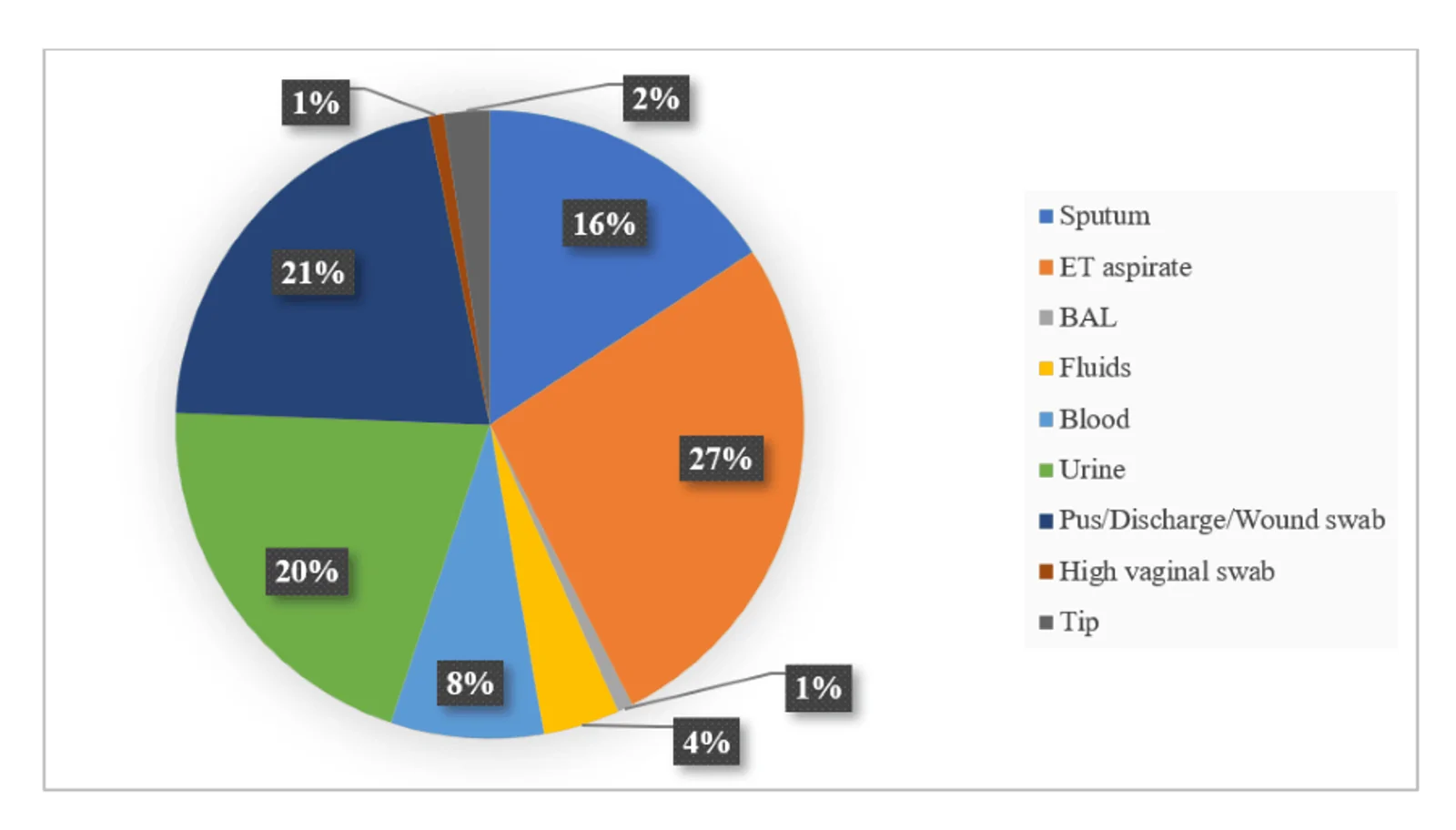
Specimen-wise distribution of NFGNB. ETA: Endotracheal aspirate; BAL: Bronchoalveolar lavage.

A total of 127 clinically significant NFGNB isolates were recovered during the study period. The majority of isolates were obtained from patients admitted to the intensive care unit (ICU), accounting for 88 (69.29%) isolates, whereas 39 (30.71%) isolates were recovered from patients in the wards (Figure 5).

**Figure 5:**
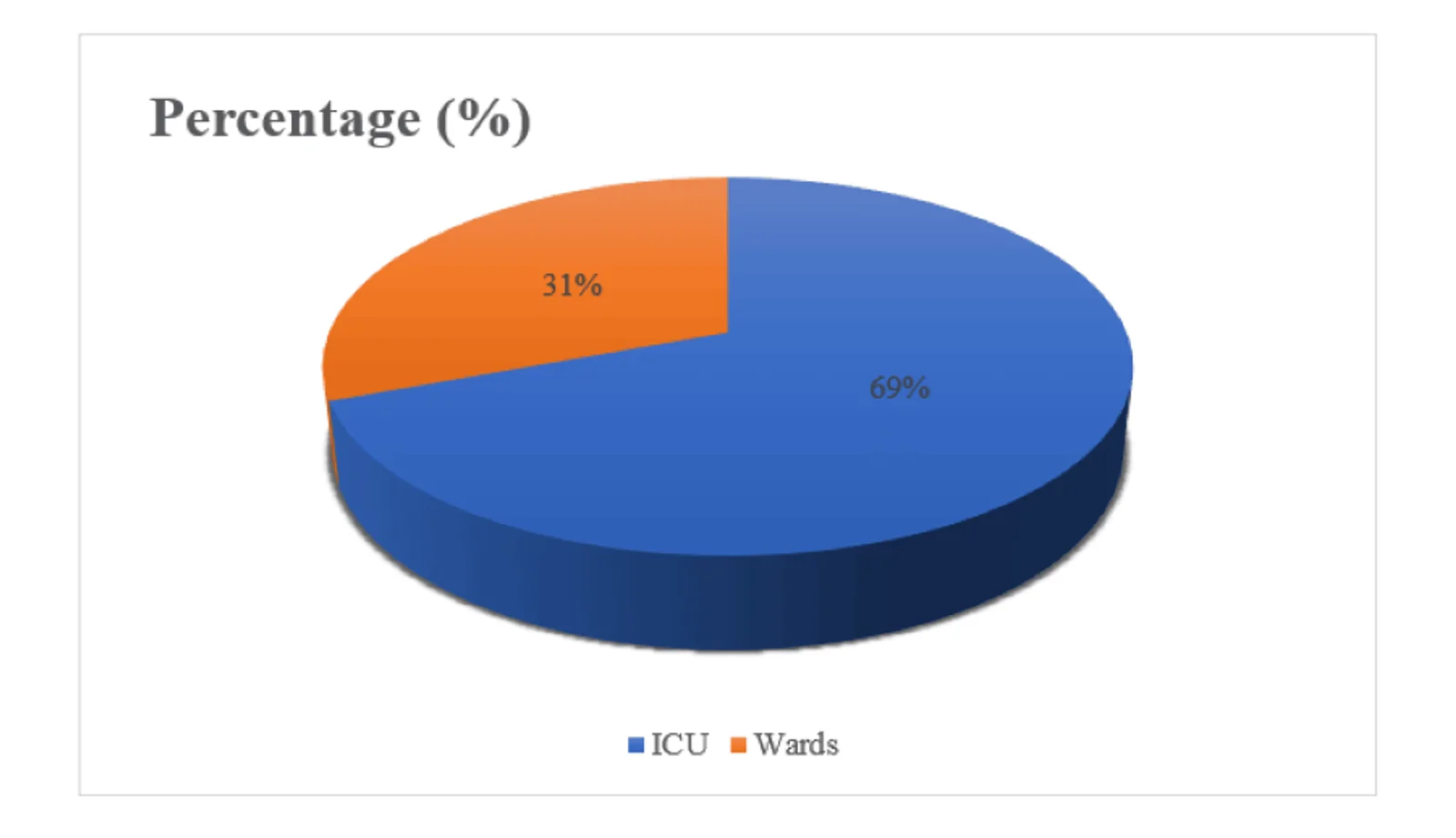
Location-wise distribution of NFGNB isolates. ICU: Intensive care unit.

Organism-Wise Antimicrobial Susceptibility Pattern

The *P. aeruginosa* isolates (n = 61) showed high resistance patterns to a number of commonly used antibiotics. Imipenem and meropenem demonstrated the highest resistance rates, with 42 (68.85%) isolates showing resistance to each antibiotic, followed by ciprofloxacin and levofloxacin, with 40 (65.57%) isolates showing resistance to each. 38 (62.30%) and 37 (60.66%) isolates showed resistance to ceftazidime and piperacillin-tazobactam, respectively. With 32 (52.46%) isolates, aztreonam showed the highest susceptibility, followed by cefepime and ceftazidime with 25 (40.98%) and 24 (39.34%) isolates, respectively. The lowest susceptibility was observed with imipenem and meropenem, with 19 (31.15%) isolates for each antibiotic. The results indicate a high degree of antimicrobial resistance in the *P. aeruginosa* isolates, especially to carbapenems and fluoroquinolones (Table 1).

**Table 1:** Antimicrobial susceptibility pattern of Pseudomonas aeruginosa (n = 61). n: Number; S: Sensitive; I: Intermediate; R: Resistant.

*Pseudomonas aeruginosa* (n = 61)						
Antibiotic	S (n)	S (%)	I (n)	I (%)	R (n)	R (%)
Piperacillin-Tazobactam	23	37.70	0	0.00	38	62.30
Ceftazidime	24	39.34	0	0.00	37	60.66
Cefepime	25	40.98	0	0.00	36	59.02
Aztreonam	32	52.46	13	21.31	16	26.23
Imipenem	19	31.15	0	0.00	42	68.85
Meropenem	19	31.15	0	0.00	42	68.85
Ciprofloxacin	21	34.43	0	0.00	40	65.57
Levofloxacin	21	34.43	0	0.00	40	65.57
Amikacin (n = 22)	5	22.73	0	0.00	17	77.27
Norfloxacin (n = 22)	6	27.27	0	0.00	16	72.73

The antimicrobial susceptibility profile of the *A. baumannii *isolates (n = 53) revealed a high level of antimicrobial resistance. The highest resistance was observed for piperacillin-tazobactam, ciprofloxacin, imipenem, meropenem, ceftazidime, and amikacin, with 46 (86.79%) isolates showing resistance to each antibiotic. Of the isolates, 45 (84.91%) were resistant to gentamicin, while 38 (71.70%) were resistant to trimethoprim-sulfamethoxazole. The highest susceptibility rates among the tested antibiotics were observed for minocycline, with 26 (49.06%) isolates, followed by doxycycline, with 25 (47.17%) isolates (Table 2).

**Table 2:** Antimicrobial susceptibility pattern of Acinetobacter baumannii (n = 53). n: Number; S: Sensitive; I: Intermediate; R: Resistant.

*Acinetobacter baumannii* (n = 53)						
Antibiotic	S (n)	S (%)	I (n)	I (%)	R (n)	R (%)
Piperacillin-Tazobactam	7	13.21	0	0.00	46	86.79
Ceftazidime	7	13.21	0	0.00	46	86.79
Imipenem	7	13.21	0	0.00	46	86.79
Meropenem	7	13.21	0	0.00	46	86.79
Doxycycline	25	47.17	8	15.09	20	37.74
Minocycline	26	49.06	6	11.32	21	39.62
Ciprofloxacin	7	13.21	0	0.00	46	86.79
Co-trimoxazole	15	28.30	0	0.00	38	71.70
Gentamicin	7	13.21	1	1.89	45	84.91
Amikacin	7	13.21	0	0.00	46	86.79
Tetracycline (n = 3)	1	33.33	0	0.00	2	66.67

The antibiotic susceptibility pattern of *S. maltophilia* isolates (n = 13) was 100% susceptible to all the antimicrobials tested. All 13 (100%) isolates were susceptible to minocycline, levofloxacin, and trimethoprim-sulfamethoxazole (Table 3).

**Table 3:** Antimicrobial susceptibility pattern of Stenotrophomonas maltophilia (n = 13). n: Number; S: Sensitive; I: Intermediate; R: Resistant.

*Stenotrophomonas maltophilia* (n = 13)						
Antibiotic	S (n)	S (%)	I (n)	I (%)	R (n)	R (%)
Minocycline	13	100	0	0	0	0
Levofloxacin	13	100	0	0	0	0
Co-trimoxazole	13	100	0	0	0	0

## Discussion

*P. aeruginosa*, *A. baumannii*, *S. maltophilia*, and the *Burkholderia cepacia* complex are the most clinically relevant NFGNB worldwide. These organisms have a broad spectrum of virulence determinants and have developed many intrinsic antimicrobial resistance mechanisms, which contribute to their success as opportunistic pathogens. The increasing prevalence of multidrug resistance, resulting from innate and acquired resistance mechanisms, is a major challenge for clinicians during the treatment and control of infections resulting from these bacteria [9,10].

The objective of this study was to determine the prevalence and distribution of NFGNB isolated from clinical specimens, to identify the predominant species of NFGNB, and to evaluate the sources of specimens involved. In addition, antimicrobial susceptibility and resistance patterns were studied for frequently employed antibacterial drugs against these pathogens. The results of the present study provide useful information for choosing appropriate empirical antibiotic therapy, improving antimicrobial stewardship programs, and strengthening infection prevention and control measures in healthcare settings in light of the increasing clinical importance and multidrug resistance of NFGNB.

The 61-70 age group had the largest burden of NFGNB infections, with 26 (20.47%) cases, followed by the 51-60 age group, with 23 (18.11%) cases, according to the findings of the present study. Similar results were reported by Sannathimmappa et al., who found the highest prevalence among patients aged >60 years, with 79 (39.3%) cases, followed by patients aged 41-60 years, with 54 (26.9%) cases. According to these findings, infections with NFGNB are more frequent in older age groups [11].

The gender-wise distribution in this study demonstrated a predominance of male patients, with 86 (67.72%) isolates obtained from males and 41 (32.28%) from females. Chavan et al. published similar results, showing that male patients had a higher distribution of NFGNB isolates (736, 59.98%) than female patients (491, 40.02%). Both studies revealed a higher prevalence of infection among males, suggesting that the burden of NFGNB infection is higher among male patients [12].

With 61 (48.03%) isolates, *P. aeruginosa* was the most prevalent NFGNB in this investigation. Followed by *A. baumannii* with 53 (41.73%) isolates and *S. maltophilia* with 13 (10.24%). Similarly, Sinha et al. reported *P. aeruginosa* as the most common isolate, with 199 (58.2%) isolates, followed by *A. baumannii* with 97 (28.4%) isolates and *S. maltophilia* with 27 (7.9%) isolates [13].

In this study, ETAs were the most common specimen source, accounting for 34 (26.77%) isolates, followed by pus/discharge/wound swabs with 27 (21.26%), urine with 26 (20.47%), sputum with 20 (15.75%), and blood with 10 (7.87%) isolates. Kumawat et al. observed a similar finding that ET aspirates obtained the highest number of NFGNB isolates, 269 (30.60%), followed by pus swabs, 168 (19.11%); blood, 142 (16.15%); and urine, 88 (10.01%). The predominance of ET aspirate specimens in both studies shows the important role of respiratory tract infections in the isolation of NFGNB [14].

In this study, 88 (69.29%) NFGNB isolates were recovered from patients admitted to the ICU, whereas 39 (30.71%) were obtained from patients admitted to the wards. Similarly, Waikhom et al. reported a higher prevalence of NFGNB isolates in the ICU, with 489 (43%) isolates, compared with 433 (38.1%) isolates from the wards [15].

The findings of the present study are comparable with those reported by Chavan et al., who also observed high resistance among *P. aeruginosa* isolates to fluoroquinolones and ceftazidime. Levofloxacin showed a significant rate of resistance in both investigations, with 40 (65.57%) isolates in this study and 470 (65.28%) isolates in the Chavan et al. study. Similarly, ceftazidime showed considerable resistance, with 37 (60.66%) and 368 (51.11%) resistant isolates, respectively. Aztreonam exhibited relatively higher susceptibility in both studies, with 32 (52.46%) susceptible isolates in the present study and 328 (45.56%) in the study by Chavan et al. [12].

Similarly, the findings of the present study are comparable with those reported by Chavan et al., who also observed high resistance among *A. baumannii* isolates to ceftazidime, ciprofloxacin, imipenem, meropenem, piperacillin-tazobactam, amikacin, gentamicin, and cotrimoxazole. Minocycline showed the highest susceptibility in both investigations, with 26 (49.06%) susceptible isolates in the current study and 241 (50.52%) in the Chavan et al. study [12].

A similar trend was observed by Chavan et al., with minocycline and trimethoprim-sulfamethoxazole demonstrating the highest antimicrobial activity against *S. maltophilia*. While Chavan et al. reported the highest susceptibility to minocycline, with eight (36.36%) susceptible isolates, and trimethoprim-sulfamethoxazole, with seven (31.82%) susceptible isolates, the current study showed 100% susceptibility to minocycline, levofloxacin, and trimethoprim-sulfamethoxazole [12].

Limitations

There are several limitations to this study. This was a single tertiary care hospital, potentially limiting the generalizability of the results to different healthcare settings. The resistance mechanisms were not molecularly characterized, and therefore, the genetic determinants of antimicrobial resistance could not be identified. However, the study provides helpful insights regarding the distribution, antimicrobial susceptibility patterns and multidrug resistance among NFGNB, which may be helpful in guiding empirical therapy, antimicrobial stewardship, and infection control practices.

## Conclusions

NFGNB, once considered contaminants, are now recognized as significant causative agents of nosocomial infections. Among the NFGNB isolated from various clinical specimens, *P. aeruginosa* was the predominant organism, followed by *A. baumannii* and *S. maltophilia*. Most of the isolates were from respiratory specimens, mainly ETAs. Infections were more common in older age groups and in male patients, indicating the clinical importance of NFGNB in hospitalized patients.

A high burden of multidrug resistance was observed in *A. baumannii *and *P. aeruginosa.* *P. aeruginosa* exhibited high resistance to imipenem, meropenem, ciprofloxacin, levofloxacin, and piperacillin-tazobactam. *A. baumannii *showed marked resistance against piperacillin-tazobactam, ceftazidime, carbapenems, fluoroquinolones, and aminoglycosides. In contrast, minocycline, levofloxacin, and trimethoprim-sulfamethoxazole were fully active against *S. maltophilia.* These findings highlight the need for ongoing antimicrobial resistance surveillance, stringent infection control practices, and evidence-based antibiotic stewardship to improve patient management and prevent the spread of MDR NFGNB.
